# Effect of Pes Anserinus Release on Postoperative Pain and Medial Stability in Medial Opening Wedge High Tibial Osteotomy

**DOI:** 10.3390/medicina62030478

**Published:** 2026-03-03

**Authors:** Han-Kook Yoon, Hyun-Cheol Oh, Joong-won Ha, Youngwoo Lee, Sang-Hoon Park

**Affiliations:** 1 Department of Orthopaedic Surgery, National Health Insurance Service Ilsan Hospital, Goyang 10444, Republic of Korea; hangugy@nhimc.or.kr (H.-K.Y.); hyuncoh@nhimc.or.kr (H.-C.O.); hjwspine@gmail.com (J.-w.H.); eclipse2085@gmail.com (Y.L.); 2 Department of Orthopedic Surgery, Severance Hospital, Yonsei University College of Medicine, Seoul 03722, Republic of Korea

**Keywords:** high tibial osteotomy, pes anserinus, medial collateral ligament, postoperative pain, knee stability

## Abstract

*Background and Objectives:* Medial opening wedge high tibial osteotomy (OWHTO) requires careful management of medial soft-tissue tension to achieve effective decompression and maintain knee stability. While superficial medial collateral ligament (sMCL) release is commonly performed, the role of pes anserinus release remains unclear. This study investigated the effect of pes anserinus release on postoperative pain, clinical outcomes, and medial stability in patients undergoing OWHTO. *Materials and Methods:* A retrospective analysis was performed on 80 knees (80 patients) that underwent OWHTO between 2012 and 2017. Patients were divided into two groups: Group A (n = 38, sMCL release only) and Group B (n = 42, sMCL + pes anserinus release). Immediate postoperative pain was assessed using visual analog scale (VAS) scores and rescue analgesic use. Clinical outcomes were evaluated with Knee Society Scores (KSSs). Radiographic medial joint opening (MJO) was measured on valgus stress radiographs preoperatively and at one year postoperatively. *Results*: Group B demonstrated significantly lower VAS pain scores at postoperative days (PODs) 1, 3, 5, 7, and 14 (*p* < 0.05) and required fewer rescue analgesics (5.5 ± 2.1 vs. 7.6 ± 3.7; *p* < 0.05). Both groups achieved comparable KSS improvement and radiographic correction (postoperative mechanical femorotibial angle: 2.1° valgus vs. 2.5° valgus). No significant intergroup or intragroup differences were observed in MJO at one-year follow-up (*p* > 0.05). *Conclusions*: Combined release of the superficial medial collateral ligament and pes anserinus during medial opening wedge high tibial osteotomy significantly reduces early postoperative pain and improves short-term functional recovery without compromising medial stability or alignment correction, although no significant long-term differences in functional outcomes or radiographic alignment were observed.

## 1. Introduction

High tibial osteotomy (HTO) is a well-established joint-preserving procedure indicated for patients with medial compartment osteoarthritis (OA) of the knee associated with varus malalignment of the lower extremity [1,2,3]. The principal goal of this procedure is to convert a varus-aligned knee into slight valgus, thereby redistributing the mechanical load from the degenerative medial compartment to the relatively preserved lateral compartment [4,5,6]. This alteration in load-bearing axis has been shown to delay the progression of medial compartment OA, reduce pain, and improve knee function in appropriately selected patients. Among the various techniques, medial opening wedge high tibial osteotomy (OWHTO) has gained wide acceptance because it allows for more precise control of the correction angle, eliminates the need for a fibular osteotomy, and avoids the risk of peroneal nerve injury that is inherent in lateral closing wedge HTO [7]. Moreover, OWHTO facilitates intraoperative assessment of alignment under fluoroscopic guidance and enables concurrent procedures such as cartilage repair or meniscal transplantation when indicated.

The medial collateral ligament (MCL) serves as the primary static restraint to valgus stress and is essential for maintaining medial joint stability [8]. Because OWHTO is performed close to the distal insertion of the superficial MCL (sMCL), medial opening of the osteotomy inevitably increases tension within the sMCL [9]. This over-tensioning may paradoxically elevate medial compartment pressure despite the intended mechanical decompression, potentially diminishing the clinical benefit of the osteotomy [6,10,11]. Previous biomechanical analyses have demonstrated that effective load transfer from the medial to the lateral compartment can be achieved only after partial or complete release of the sMCL [6,11,12]. However, excessive release may lead to undesirable valgus laxity, resulting in impaired postoperative joint stability [13]. Therefore, meticulous regulation of MCL tension is essential to achieve an optimal balance between reducing medial contact pressure and preserving valgus stability. Therefore, careful modulation of MCL tension during OWHTO is essential to achieve an optimal balance between medial decompression and preservation of valgus stability. Understanding the biomechanical and clinical implications of MCL handling in OWHTO is critical for optimizing surgical outcomes and minimizing complications related to instability or inadequate correction.

The pes anserinus, formed by the conjoined tendons of the sartorius, gracilis, and semitendinosus muscles, also contributes to medial knee stability by providing dynamic anteromedial support [14]. Functioning synergistically with the medial collateral ligament (MCL), the pes anserinus resists valgus and external rotational stresses, particularly in mid-flexion, thereby contributing to overall medial soft-tissue integrity [14]. During OWHTO, the pes anserinus is located directly over the osteotomy site and thus requires intraoperative management. Surgeons commonly choose between two approaches: subperiosteal elevation or in situ preservation. Preserving its native insertion may help maintain dynamic stability after the osteotomy and potentially prevent excessive valgus laxity. However, tension within the pes anserinus tendons may increase following the bony opening, like that of the superficial MCL, potentially limiting the intended decompression of the medial compartment and diminishing early postoperative pain relief. Retaining its native attachment may sustain medial stability but can increase tensile stress similar to the sMCL, potentially limiting pressure reduction in the medial compartment [15]. Conversely, releasing the pes anserinus during OWHTO can facilitate adequate medial opening and may contribute to a greater reduction in medial compartment contact pressure. Nevertheless, this release may also disrupt the dynamic stabilizing function of the pes anserinus, possibly resulting in increased medial laxity or altered joint mechanics during gait. Thus, the decision regarding pes anserinus management involves a trade-off between achieving optimal load redistribution and preserving medial soft-tissue stability.

Achieving an appropriate balance between medial soft-tissue tension, compartmental contact pressure, and overall joint stability is thus critical for successful OWHTO. Given this interplay, further clarification of the biomechanical and clinical consequences of pes anserinus release is warranted. Accordingly, this study aimed to investigate the impact of pes anserinus release on immediate postoperative pain, functional outcomes, and medial knee stability in patients undergoing OWHTO, focusing particularly on the interplay between medial compartment pressure and structural stability.

## 2. Materials and Methods

### 2.1. Patients

Ethical approval for this study was obtained from the institutional review board of our hospital. A retrospective review was conducted on patients who underwent medial opening wedge high tibial osteotomy (OWHTO) between 2012 and 2017. The indications for OWHTO included: (1) isolated medial compartment osteoarthritis (OA), (2) varus malalignment greater than 3° and less than 20°, (3) age younger than 65 years, (4) a near-normal range of motion, (5) intact lateral and patellofemoral compartments, and (6) the desire to maintain a high postoperative activity level.

The inclusion criteria for the present study were patients aged 40 to 65 years who underwent medial OWHTO using a locking compression plate and had a minimum follow-up of 12 months. Exclusion criteria included the presence of lateral or patellofemoral compartment OA, inflammatory arthropathy, simultaneous bilateral OWHTO, or prior surgery on the affected knee.

During the study period, 90 knees (88 patients) underwent OWHTO. Eight patients were excluded: five were lost to follow-up, two underwent simultaneous bilateral procedures, and one had a prior knee operation. Finally, 80 knees (80 patients) were included in the analysis. Baseline demographic data were collected from medical records. All patients underwent standard weight-bearing anteroposterior long-leg radiographs and magnetic resonance imaging (MRI) preoperatively.

A single orthopedic surgeon performed all procedures at a single institution following a standardized protocol. Among the 80 patients, group A comprised 38 knees that underwent OWHTO with sMCL release only, whereas group B included 42 knees that underwent both sMCL and pes anserinus release.

Spinal anesthesia was administered using 8–12 mg of 0.5% bupivacaine to achieve a T8–T10 level sensory block. Surgical steps, anesthetic methods, and postoperative rehabilitation protocols were standardized across all patients. Passive range of motion exercises and toe-touch weight-bearing with crutches were allowed immediately after surgery. Partial to full weight-bearing was permitted at 2 weeks, and return to pivoting or impact sports was allowed following radiographic confirmation of bone union.

### 2.2. Surgical Technique

Preoperative planning was based on standard weight-bearing anteroposterior long-leg radiographs. The correction angle was calculated using the Miniaci method, targeting an alignment passing through the Fujisawa point. Diagnostic knee arthroscopy was performed before osteotomy to assess intra-articular cartilage condition and to treat concomitant lesions such as medial meniscus tears or focal cartilage defects. Under tourniquet control, an anteromedial oblique incision was made to expose the proximal tibia. In group A, the superficial medial collateral ligament (sMCL) was subperiosteally detached entirely from its tibial insertion, while the pes anserinus was preserved. In group B, both the sMCL and pes anserinus were fully released. Two guide wires were inserted toward the lateral cortex, approximately 1 cm proximal to the fibular head, under fluoroscopic guidance. The posterior tibial surface was carefully exposed using a periosteal elevator, and a retractor was placed to protect the popliteal vessels. An oblique osteotomy was created with an oscillating saw, preserving the lateral cortex. A secondary vertical osteotomy behind the tibial tubercle was added for a biplanar configuration. Gradual opening of the osteotomy site was performed using flat chisels to prevent excessive posterior tibial slope increase. The desired correction angle was achieved using a laminar spreader and confirmed radiographically. Fixation was accomplished in all cases with a TomoFix™ locking plate (Synthes, Solothurn, Switzerland), and the osteotomy gap was filled with demineralized bone matrix (DBM).

### 2.3. Postoperative Management

Continuous passive motion was initiated on postoperative day (POD) 1 within a pain-tolerable range. No braces or immobilizers were applied. Crutch-assisted ambulation was permitted from the first postoperative day depending on patient tolerance. Full weight-bearing was restricted for the initial 4 weeks. For analgesia, all patients received intravenous patient-controlled analgesia (IV-PCA) containing fentanyl and ramosetron. In cases of breakthrough pain, intramuscular tramadol (50 mg) was administered as rescue medication. All patients were discharged on POD 14.

### 2.4. Evaluation

Postoperative pain was evaluated using a visual analog scale (VAS; 0 = no pain, 10 = worst imaginable pain) at POD 1, POD 3, POD 5, POD 7, POD 14, and 4 weeks postoperatively. The patients were evaluated at 3 months postoperatively and at annual intervals thereafter. The total number of rescue analgesics administered during hospitalization was also recorded. Clinical outcomes were assessed using the Knee Society Score. Postoperative pain was evaluated using a visual analog scale (VAS; 0 = no pain, 10 = worst imaginable pain) at POD 1, POD 3, POD 5, POD 7 and POD 14 postoperatively. The patients were evaluated at 1 month and 3 months postoperatively, and at annual intervals thereafter. The total number of rescue analgesics administered during hospitalization was also recorded. Clinical outcomes were assessed using the Knee Society Score (KSS) preoperatively and at 1-year follow-up.

Radiologic outcomes were evaluated on weight-bearing anteroposterior long-leg radiographs obtained preoperatively and at 1 year after surgery. The mechanical femorotibial angle (mFTA) was defined as the angle between the mechanical axes of the femur and tibia.

Medial knee laxity was quantified by measuring the medial joint space opening (MJO) on Telos valgus stress radiographs with the knee in full extension, both preoperatively and at 1 year postoperatively. The MJO measurement method followed that described by Kennedy and Fowler. A horizontal line was drawn tangential to the most distal subchondral surfaces of both femoral condyles, and another line was drawn along the most proximal subchondral surface of the tibial condyles. The vertical distance between these two lines in the medial compartment represented the MJO value. All radiographic measurements were performed by two independent observers (YHK and PSH) who were blinded to patient clinical data.

### 2.5. Statistical Analysis

Normal data distribution was examined, and appropriate tests were applied. Student’s t-test, Mann–Whitney U test, and Fisher’s exact test were used to compare demographic and outcome variables between groups. Independent *t*-tests confirmed the absence of significant baseline differences. Gender distribution showed no association (odds ratio = 1.15, *p* = 1.00) according to Fisher’s exact test. Clinical and radiographic parameters were analyzed using the Mann–Whitney U test. All statistical analyses were performed using SPSS version 23.0 software (SPSS Inc., Chicago, IL, USA), and statistical significance was defined as *p* < 0.05.

## 3. Results

### 3.1. Patient Demographics

The demographic characteristics of both groups are summarized in Table 1. Group A comprised 38 patients (35 women and 3 men), and Group B comprised 42 patients (35 women and 7 men). The mean age at the time of surgery was 55.1 ± 7.2 years in Group A and 57.7 ± 3.9 years in Group B. The mean body mass index (BMI) was 26.4 ± 3.2 kg/m^2^ in Group A and 26.1 ± 3.6 kg/m^2^ in Group B. There were no statistically significant differences between the two groups in terms of age, sex distribution, or BMI (*p* > 0.05).

### 3.2. Clinical Outcome

The clinical outcomes are presented in Table 2 and Table 3. Group A demonstrated consistently higher mean visual analog scale (VAS) pain scores than Group B at all postoperative time points. The differences in mean VAS scores at postoperative days (PODs) 1, 3, 5, 7, and 14 were statistically significant (*p* < 0.05). Group B demonstrated significantly lower VAS pain scores at postoperative days (PODs) 1, 3, 5, 7, and 14 (*p* < 0.05) and required fewer rescue analgesics (5.5 ± 2.1 vs. 7.6 ± 3.7; *p* < 0.05). By postoperative month 3, the mean pain score had decreased in both groups, and the intergroup difference was no longer significant (*p* = 0.555).

The mean total number of rescue analgesic administrations during hospitalization was significantly higher in Group A than in Group B (7.6 ± 3.7 vs. 5.5 ± 2.1, respectively; *p* < 0.05).

The mean Knee Society Scores (KSSs) improved significantly in both groups from the preoperative assessment to the 1-year follow-up, indicating substantial functional recovery. However, no statistically significant difference was found between the two groups at either the preoperative or 1-year evaluation (*p* > 0.05).

Table 4 summarizes total knee arthroplasty (TKA) conversion rates after high tibial osteotomy (HTO), comparing sMCL release (Group 1, n = 38) to dual sMCL + pes anserinus release (Group 2, n = 42). Verified *p*-values via Fisher’s exact test confirm no significant differences, supporting noninferiority at 5-year follow-up.

### 3.3. Radiographic Outcome

The radiographic results are summarized in Table 5. No statistically significant differences were observed in radiographic parameters between the two groups at either the preoperative or postoperative assessment (*p* > 0.05).

The mean preoperative mechanical femorotibial angle (mFTA) was 8.6° ± 2.4° of varus (range, 3.7–11.3°) in Group A and 8.9° ± 2.8° of varus (range, 4.5–14.3°) in Group B. Following osteotomy, the mean mFTA improved to 2.1° ± 1.2° of valgus (range, 0–5.2°) in Group A and 2.5° ± 1.4° of valgus (range, 0.5–5.9°) in Group B.

The mean medial joint opening (MJO) in Group A was 6.4 ± 1.4 mm preoperatively and 6.5 ± 0.8 mm at the 1-year follow-up. In Group B, the mean MJO values were 6.3 ± 2.1 mm preoperatively and 6.3 ± 1.5 mm at 1 year. There were no significant changes in MJO from preoperative to postoperative assessments in either group (*p* = 0.57 and *p* = 0.88, respectively).

These findings indicate that while combined sMCL and pes anserinus release effectively reduced early postoperative pain without compromising medial stability, it did not influence overall correction accuracy or radiographic outcomes.

## 4. Discussion

The primary finding of this study was that additional release of the pes anserinus during medial opening wedge high tibial osteotomy (OWHTO) significantly reduced early postoperative pain and facilitated early functional recovery without increasing medial laxity in the long term. Patients who underwent combined release of the superficial medial collateral ligament (sMCL) and the pes anserinus reported substantially lower visual analog scale (VAS) pain scores during the initial two postoperative weeks and achieved higher Knee Society Scores (KSSs) up to three months after surgery compared with those who underwent isolated sMCL release. These results indicate that the pes anserinus release can contribute to enhanced early recovery while maintaining joint stability.

The effect of medial soft-tissue tension on joint pressure and stability after OWHTO has been well documented. The pes anserinus is located directly over the osteotomy site and therefore requires deliberate intraoperative management during medial opening wedge high tibial osteotomy (OWHTO). Surgeons generally adopt one of two approaches: subperiosteal elevation of the pes anserinus or its preservation in situ. Maintaining its native insertion may provide dynamic stabilization and prevent excessive valgus laxity in the early postoperative period. However, as the osteotomy gap is gradually widened, tensile stress within the pes anserinus tendons can increase, similar to the superficial medial collateral ligament (sMCL), potentially impeding complete decompression of the medial compartment and diminishing the desired reduction in intra-articular pressure and early postoperative pain relief. Several biomechanical investigations have demonstrated that sMCL release is critical for achieving adequate decompression of the medial compartment after wedge opening [6,11,12]. Agneskirchner et al. reported that OWHTO performed without MCL release significantly increased medial contact pressure, whereas complete sMCL release produced a marked reduction in medial load following wedge distraction [6]. Similarly, Van Egmond et al. observed a significant shift in load from the medial to the lateral compartment only after complete sMCL release [11]. Van Egmond et al. demonstrated that only after complete sMCL release did a significant shift in the tibiofemoral load from the medial to the lateral compartment occur. Beyond decreasing intra-compartmental pressure, sMCL release also improves surgical exposure of the osteotomy plane and reduces the risk of posterior neurovascular injury by facilitating controlled wedge distraction [11]. In addition to relieving pressure, sMCL release facilitates exposure of the osteotomy plane and helps prevent posterior neurovascular injury [16].

However, as the sMCL is the primary restraint to valgus stress, its release introduces a theoretical risk of valgus instability, given its fundamental role as the primary static restraint to valgus stress [17]. Van Egmond et al. reported a mean increase of 7.9° in valgus laxity after complete release in cadaveric knees [11]. Pape et al. emphasized the importance of preserving the anterior fibers of the sMCL to maintain valgus stability and recommended minimal release whenever possible [10]. To address this concern, several authors have proposed sMCL repair or reattachment techniques to restore medial tension following osteotomy. Nonetheless, in vivo clinical data do not consistently demonstrate postoperative valgus instability. Long-term clinical studies have generally failed to demonstrate a meaningful increase in valgus laxity after OWHTO when proper surgical technique and postoperative rehabilitation are applied. Woodacre et al. followed 115 OWHTO patients for a mean of 8.4 years and found no clinically relevant valgus instability [18]. Similarly, Han et al. (n = 209) reported a 29.7% overall complication rate without any instance of valgus laxity after OWHTO.

Several authors have also described spontaneous recovery of medial stability after sMCL release, suggesting that postoperative soft-tissue remodeling may compensate for the temporary laxity observed immediately after osteotomy. Seo et al. found that although the medial joint opening (MJO) increased immediately after release, it returned to near-baseline values after plate fixation, likely due to tensioning of adjacent medial structures [13,19]. Sato et al. also observed recovery of laxity one year after surgery, attributing it to the healing of the sMCL and compensatory tensioning of nearby medial stabilizers [14]. These findings collectively imply that transient valgus laxity following sMCL release may be a self-limiting condition that resolves with tissue healing, fibrosis, and gradual reestablishment of medial soft-tissue tension over time. This adaptive process underscores the importance of controlled rehabilitation, which allows musculoskeletal reconditioning and scar remodeling to occur without overloading the healing structures.

While prior studies have focused predominantly on the role of the sMCL, relatively few have investigated the biomechanical or clinical function of the pes anserinus. The pes anserinus, composed of the conjoined tendons of the sartorius, gracilis, and semitendinosus muscles, acts as a dynamic medial stabilizer, particularly in early postoperative phases before the sMCL regains its original tension [20]. Our results demonstrated that combined release of both the sMCL and pes anserinus improved early pain and function without radiographic evidence of long-term medial laxity. This observation aligns with the findings of Nakamura et al., who suggested that preservation of the pes anserinus might increase tension around the osteotomy site, transmitting compressive force across the bone graft and thereby increasing medial compartment pressure. The tension within the pes anserinus may also compress the osteotomy gap, limit medial decompression, and contribute to immediate postoperative pain.

In contrast, Kim et al. reported that when the pes anserinus was repaired beneath the fixation plate after combined release, joint stability and alignment were well maintained, with no radiographic difference in the joint line convergence angle (JLCA) before and after plate removal [21]. In our study, even without repair, we observed no significant difference in medial joint opening one year after OWHTO, suggesting that any short-term loss of tension from pes anserinus release may be compensated by healing and by tension within other medial structures as the osteotomy site consolidates.

The principal favorable finding of this study was the reduction in pain during the early postoperative period. Although this difference was statistically significant, the effect appeared to be limited to the initial postoperative phase and diminished over time. Therefore, the clinical significance of this result should not be overstated. The present findings suggest that, while short-term benefits in postoperative pain relief may exist, there are no apparent long-term differences in knee function or alignment between the groups.

Although pes anserinus release appears to facilitate early pain relief and soft-tissue decompression, several considerations should temper its routine use. First, complete detachment theoretically reduces dynamic medial resistance and may increase the risk of valgus laxity if excessive. Second, as Nakamura et al. pointed out, the preserved pes anserinus may aid compression at the osteotomy site, which could promote bone union and reduce the risk of plate failure in cases of unstable lateral hinge fracture [20]. Thus, selective, tension-adjusted management of the pes anserinus—rather than a universally applied release—may provide the most favorable balance between pain control, stability, and healing.

There are several limitations to the present study. The retrospective design carries inherent risks of selection and recall biases. During the initial 2.5 years of the study period, patients underwent the Group A surgical technique, after which, the Group B procedure was adopted. Therefore, patient allocation was determined by the chronological sequence of surgical practice rather than by randomization, which may have introduced an element of selection bias. The sample size was relatively small, and the follow-up duration was limited to five years, which precludes evaluation of late-onset valgus instability or long-term functional differences. Owing to the relatively short duration of follow-up, the long-term outcomes, particularly those related to conversion to total knee arthroplasty during the subsequent clinical course, could not be fully assessed in this study. As such, the findings should be interpreted with caution, and extended longitudinal follow-up with comprehensive evaluation of conversion rates and prosthesis-related outcomes will be necessary to validate the durability and long-term clinical efficacy of the present surgical approach. Medial stability was assessed using the medial joint opening (MJO) measured on valgus stress radiographs obtained preoperatively and at one year postoperatively. Although this method is widely accepted, it does not reflect early postoperative valgus laxity, which may be particularly relevant considering the extent of medial soft-tissue release performed. The lack of early or dynamic assessment of medial stability should be acknowledged as a study limitation, and interpretations regarding the preservation of medial stability should therefore be made with caution. Immediate postoperative valgus laxity was not measured dynamically; thus, the in vivo behavior of medial soft-tissue tension shortly after release could not be fully characterized. Additionally, individual variations in osteotomy correction angle, bone quality, and rehabilitation compliance were not controlled. Larger, prospective studies will be required to validate the long-term safety and biomechanical effects of pes anserinus release during OWHTO.

## 5. Conclusions

Simultaneous release of the superficial medial collateral ligament and pes anserinus during medial opening wedge high tibial osteotomy effectively alleviates early postoperative pain and enhances early functional recovery, without increasing medial joint laxity or compromising radiographic alignment. Although long-term outcomes remain comparable to those achieved with isolated sMCL release, selective pes anserinus release offers a practical advantage in improving early postoperative comfort and rehabilitation compliance. Combined release of the superficial medial collateral ligament and pes anserinus during medial opening wedge high tibial osteotomy significantly reduces early postoperative pain and improves short-term functional recovery without compromising medial stability or alignment correction, although no significant long-term differences in functional outcomes or radiographic alignment were observed. Careful intraoperative modulation of medial soft-tissue tension remains essential to preserving the balance between stability and pressure redistribution in OWHTO.

## Figures and Tables

**Table 1:** Demographic factors.

Parameters	Group 1 sMCL Release (n = 38)	Group 2 Both sMCL & Pes Anserinus Release (n = 42)	*p*-Value
Age (years)	55.1 ± 7.2	57.7 ± 3.9	0.52
Gender (female/male)	35/3	35/7	1.0
Body mass index (kg/cm^2^)	26.4 ± 3.2	26.1 ± 3.6	0.35

Data are presented as means with standard deviation. *p*-value < 0.05.

**Table 2:** Postoperative changes in the VAS score.

Postoperative Day	Group 1 sMCL Release (n = 38)	Group 2 Both sMCL & Pes Anserinus Release (n = 42)	*p*-Value
Preop.	9.3 (7–10)	9.4 (4–10)	0.712
Postop 1 day	9.6 (7–10)	7.1 (4–10)	<0.0001
1 week	7.8 (3–0)	5.7 (3–9)	<0.0001
1 mos	6.6 (0–8)	5.5 (1–9)	0.016
3 mos	4.4 (0–7)	4.2 (0–5)	0.555
1 yr	2.1 (0–6)	2.1 (0–4)	1.00
2 yrs	2.8 (0–8)	2.7 (0–9)	0.834
3 yrs	3.1 (0–9)	3.0 (0–7)	0.824
5 yrs	3.4 (0–10)	3.3 (0–10)	0.858

Visual Analog Scale (VAS).

**Table 3:** Postoperative changes in the KSS score.

Postoperative Day	Group 1 sMCL Release (n = 38)	Group 2 Both sMCL & pes Anserinus Release (n = 42)	*p*-Value
Preop.	66.5 ± 6.9	65.1 ± 7.1	0.375
1 mos	79.5 ± 8.3	85.5 ± 9.8	0.004
3 mos	81.1 ± 8.8	89.4 ± 6.1	<0.0001
1 yr	91.1 ± 6.5	90.7 ± 5.1	0.759
2 yrs	84.2 ± 8.1	84.4 ± 7.5	0.909
3 yrs	81.9 ± 8.1	82.8 ± 8.2	0.623
5 yrs	78.8 ± 8.3	79.2 ± 8.4	0.831

**Table 4:** Total knee arthroplasty conversion cases after HTO surgery.

	Group 1 sMCL Release (n = 38)	Group 2 Both sMCL & Pes Anseinus Release (n = 42)	
postop 1 yr	0	0	1.00
postop 2 yrs	0	0	1.00
postop 3 yrs	1	0	0.475
postop 4 yrs	3	2	0.664
postop 5 yrs	5	3	0.467
total	9	5	0.239

**Table 5:** Postoperative changes in radiographic parameters.

Parameter	Group 1 sMCL Release (n = 38)	Group 2 Both sMCL & Pes Anserinus Release (n = 42)	*p*-Value
Preop. mFTA (varus)	8.6 ± 2.4 (3.7–11.3)	8.9 ± 2.8 (4.5–14.3)	>0.05
Postop. mFTA (valgus)	2.1 ± 1.2 (0–5.2)	2.5 ± 1.4 (0.5–5.9)	>0.05
Preop MJO (mm)	6.4 ± 1.4	6.3 ± 2.1	>0.05
1 yr MJO (mm)	6.5 ± 0.8	6.3 ± 1.5	>0.05
MJO change *p*-value	0.57	0.88	

mFTA (mechanical femoro-tibial angle), MJO (medial joint space opening).

## Data Availability

The datasets used and analyzed during the current study are available from the corresponding author upon reasonable request.
